# Genetic Dissection of Early Blight Resistance in Tetraploid Potato

**DOI:** 10.3389/fpls.2022.851538

**Published:** 2022-03-25

**Authors:** Weiya Xue, Kathleen G. Haynes, Christopher R. Clarke, Xinshun Qu

**Affiliations:** ^1^Department of Plant Pathology and Environmental Microbiology, The Pennsylvania State University, University Park, PA, United States; ^2^Genetic Improvement of Fruits and Vegetables Laboratory, US Department of Agriculture Agricultural Research Service, Beltsville, MD, United States

**Keywords:** potato, early blight, maturity, broad sense heritability, linkage map, QTL

## Abstract

Early blight, caused by the fungus *Alternaria solani*, is one of the most economically important diseases of potatoes worldwide. We previously identified a tetraploid potato clone, B0692-4, which is resistant to early blight. To dissect the genetic basis of early blight resistance in this clone, a full-sib tetraploid potato population including 241 progenies was derived from a cross between B0692-4 and a susceptible cultivar, Harley Blackwell, in this study. The population was evaluated for foliage resistance against early blight in field trials in Pennsylvania in 2018 and 2019 and relative area under the disease progress curve (rAUDPC) was determined. The distribution of rAUDPC ranged from 0.016 to 0.679 in 2018, and from 0.017 to 0.554 in 2019. Broad sense heritability for resistance, as measured as rAUDPC, was estimated as 0.66–0.80. The population was also evaluated for foliar maturity in field trials in Maine in 2018 and 2020. A moderate negative correlation between rAUDPC and foliar maturity was detected in both years. A genetic linkage map covering a length of 1469.34 cM with 9124 SNP markers was used for mapping quantitative trait loci (QTL) for rAUDPC and foliar maturity. In 2018, three QTLs for early blight were detected; two of them on chromosome 5 overlapped with QTLs for maturity, and one of them on chromosome 7 was independent of maturity QTL. In 2019, six QTLs for early blight were detected; two QTLs on chromosome 5 overlapped with QTLs for maturity, and the other four QTLs did not overlap with QTLs for maturity. The identification of these QTLs provides new insight into the genetic basis of early blight resistance and may serve as sources for marker-assisted selection for early blight resistance breeding.

## Introduction

Potato (*Solanum tuberosum* L.) is one of the most important food crops in the world. Early blight, caused by fungus *Alternaria solani*, is one of the major pathogen stresses limiting potato production, especially in regions with high temperatures (Holley et al., 1985; Edin and Andersson, 2014; Runno-Paurson et al., 2015; Tymon et al., 2016). Yield losses by early blight can reach up to 40% without fungicide treatments (Harrison and Venette, 1970; Teng and Bissonnette, 1985). Although fungicide application is the most efficient way to control early blight in potatoes (Yellareddygari et al., 2019), its use causes concerns about environmental contamination and food safety. Repeated application of fungicides also leads to the emergence of fungicide resistance in *A. solani* (Wharton et al., 2012; Gudmestad et al., 2013).

Genetic disease resistance is the best long-term solution for sustainable management of early blight. However, only a few studies have been conducted to identify early blight resistant germplasm. Boiteux et al. (1995) screened 934 potato breeding clones and cultivars in Brazil, only 27 clones were identified that had less lesioned leaf area than the resistant standard cultivar. Abuley et al. (2018) found 12 “slow blighting” cultivars among 38 cultivars in Denmark. Xue et al. (2019) screened 217 old and new commercial cultivars, only 29 cultivars clustered into the resistant group. Genetic resistant resources have also been found in wild *Solanum* species. Jansky and Rouse (2003) found a few early blight resistant clones among 32 potato clones from interspecific hybrids. *S. neorossii*, *S. commersonii*, and *S. tarijense* were identified as the most early blight resistant species from 156 accessions among 41 wild relatives of potato by Jansky et al. (2008). In these previous studies, resistance levels showed a continuous distribution, suggesting that there were no dominant resistance genes for early blight resistance in these populations. However, Wolters et al. (2021) recently found early blight dominant genetic resistance from the cross between *S. commerosum* subp. *malmeanum* and diploid *S. tuberosum*. Early blight resistance in potatoes is highly heritable. Christ and Haynes (2001) estimated broad sense heritability of early blight as 0.73 and narrow sense heritability as 0.61 in a diploid random-mated hybrid population of *S. phureja* × *S. stenotomum*. In another study, Ortiz et al. (1993) estimated narrow sense heritability as 0.64–0.78. In an open-pollinated 4x-2x hybrid population, broad sense heritability was estimated as 0.91 (Christ et al., 2002). Xue et al. (2019) estimated broad sense heritability as 0.89 in 217 tetraploid cultivars. In both diploid or tetraploid potato germplasm, broad sense and narrow sense heritability were high, and resistance could be introgressed from diploid to tetraploid potato (Christ et al., 2002). Only partial resistant cultivars or clones were found in previous studies and progress in early blight resistant breeding has therefore been slow.

Research on the genetic basis of early blight resistance in potato is limited (Zhang, 2005; Odilbekov et al., 2020). Odilbekov et al. (2020) investigated tuber resistance and foliar resistance in a tetraploid potato population. Tuber resistant quantitative trait locus (QTLs) were mapped on chromosomes 1, 2, 3, 4, 8, 11, and 12. Foliar resistant QTLs were mapped on chromosomes 1, 5, 6, 7, 11, and 12. Zhang (2005) mapped five QTLs for foliar resistance on chromosomes 4, 5, 9, 11, and 12 in a diploid population. However, there were limitations in these studies due to incomplete linkage maps and/or the small size of the segregating mapping population.

Like late blight resistance, early blight resistance is also correlated with late maturity (Johanson and Thurston, 1990; Visker et al., 2003; Xue et al., 2019). Usually, early-maturing cultivars were more susceptible, and later-maturing cultivars were more resistant. Maturity correlated resistance is usually not considered as true resistance. It could be one of the effects of life-cycle difference. In some cases, potato plants can escape the infections by early maturing. Meanwhile, lateness is not a desirable trait in potato breeding, especially in short growth season regions. To further dissect non-maturity-based resistance and maturity-based resistance, maturity corrected resistance was applied in early blight research through the linear regression of maturity on rAUDPC. By comparing the difference between the predicted value and the observed value of rAUDPC, Xue et al. (2019) found genetic components of early blight resistance unrelated to late maturity in 7 of the 217 cultivars studied. Zhang (2005) initiated a QTL mapping study for early blight resistance in a diploid potato population. Two QTLs mapped on chromosomes 4 and 5 overlapped with QTLs for maturity. The other three QTLs on chromosomes 9, 11, and 12 were unlinked with maturity. Recently, Odilbekov et al. (2020) investigated early blight resistance and leaf defoliation (foliage maturity) in a tetraploid population. Two early blight resistant QTLs on chromosomes 5 and 11 were mapped independent of foliage maturity.

The genetic determination of early blight resistance needs additional studies because of the small population sizes and incomplete linkage maps in previous studies (Zhang, 2005; Odilbekov et al., 2020). Zhang (2005) used 170 AFLP and 47 SSR markers for linkage map construction. Odilbekov et al. (2020) used 10k SNP markers for linkage map construction. With the development of high-density SNP marker linkage maps, QTL mapping has become more convenient in potato research. In the past 10 years, we identified an early blight resistant tetraploid potato clone B0692-4. We hypothesized that disease-resistant genes/QTLs exist in this clone. In this study, we produced a population from a cross between B0692-4 and an early blight susceptible cultivar. We used 20K SNP markers for linkage map construction (Hamilton et al., 2011). We developed a high-density linkage map to dissect early blight resistance in the tetraploid potato population and QTLs for early blight resistance were identified.

## Materials and Methods

### Plant Material and Crossing

A population from a cross of Harley Blackwell (♀), which is susceptible to early blight, and B0692-4 (♂) which is resistant to early blight, was made in 2014. True seeds were surface sterilized and placed in individual test tubes of MS media and germinated in the USDA/ARS tissue culture facilities in Beltsville, MD. F1 seedlings (genotypes) were micropropagated to generate four plantlets per genotype and when the plantlets had a few leaves and sufficient root system, these were transplanted into the greenhouses in Beltsville beginning 26 July 2015 and continuing for several months until all genotypes were planted. Minitubers were harvested from each genotype beginning 30 November 2015 and harvest terminated on 23 February 2016. Where available, 12 minitubers per genotype were planted in the field on Chapman Farm, Mapleton, Maine on 25 May 2016. At harvest, 13 September 2016, all tubers were harvested. All the genotypes were then propagated each year on Chapman Farm, Mapleton, Maine.

Two hundred and forty-one F1 genotypes from the segregating population and their parents were planted in the field on Chapman Farm on 23 May 2017 using an augmented design with two replications. Twenty seed pieces were planted for each genotype. Tubers were harvested 11-12 September 2017 and divided between USDA/ARS and The Pennsylvania State University (PSU). Genotypes from the segregating population and their parents were planted in the field on Chapman Farm on 17–18 May 2018 in a randomized complete block design with two replications of 20 plants per genotype in each plot. Due to weather and other constraints, plants were harvested 10, 12, 25, 28 September 2018 and divided between USDA/ARS and PSU.

### Field Early Blight Evaluation Experiments

Early blight resistance evaluation experiments were conducted at The Pennsylvania State University Russell E. Larson Agricultural Research Center in Pennsylvania Furnace, Pennsylvania in 2018 and 2019. Two parents and 241 full-sib F1 progenies were planted in a randomized complete block design with two replicates on 7 June 2018 and 4 June 2019. Plots consisted of a single 121.9-cm long row with five seed pieces planted in each plot, with a 121.9-cm break between plots. Each entry had an adjacent row of the susceptible cultivar Dark Red Norland as a spreader row. Commercial potato management practices were followed throughout the growing season, without fungicides for early blight. To promote the uniform spread of the pathogen to all treatment plots, spreader rows were spray-inoculated with a conidial mixture of two isolates of *Alternaria solani*, at a concentration of 1.75 × 10^4^ conidia/ml on 30 July 2018 and 7.5 × 10^4^ on 25 July 2019. Early blight disease severity was visually assessed by estimating the percentage of diseased foliage on a 0 to 100% scale on each F1 progeny and parents plots every 5–7 days according to weather conditions. In 2018, assessments were made on 9, 16, 23, 29 August and 5, 11 September. In 2019, assessments were made on 12, 19, 25, 29 August and 3 September. Area under the disease progress curve (AUDPC) was calculated as described by Shaner and Finney (1977). The final early blight resistance rating was represented by relative disease progress curve (rAUDPC) which was calculated by dividing the AUDPC by 100 times the duration of disease assessment days (Fry, 1978).

### Field Maturity Evaluation Experiments

Maturity evaluation experiments were conducted on Chapman Farm, Mapleton, Maine in 2018 and 2020. Two parents and 241 full-sib F1 progenies were planted in a randomized complete block design with two replicates on 17–18 May 2018 and 21 May 2020. Each plot contained 20 plants. Standard crop management practices were followed throughout the growing season. On 15 August 2018 and 12 August 2020, leaf senescence (foliage maturity) per plot was visually assessed. The maturity was scored into 6 levels: 1 = very early maturity; 2 = early maturity; 3 = medium early maturity; 4 = medium late maturity; 5 = late maturity; and 6 = very late maturity. The final values of maturity were the means of 2 years’ data because of no significant interaction between year and genotype for maturity.

### Genotypic Data Collection

Plant leaf samples were collected from young plants in the field. DNA was isolated from leaves by Qiagen DNeasy Plant Mini Kit following the manufacturer’s protocol. DNA quality was checked on 1% agarose gel and Nanodrop. DNA (300 ng) from each genotype was used for SNP genotyping by SolCAP potato array (v3) at Michigan State University (Hirsch et al., 2014). SolCAP potato array (v3) contains about 20k SNP on the whole potato genome. The position of each marker on the chromosome was based on the potato reference genome, i.e., pseudomolecules v4.03.

### Statistical Analysis

The rAUDPC data were square-root transformed for the analysis of variance. Due to homogeneous error variances, the data were subsequently combined over years for analysis with the STAR R package (IRRI, 2014). The ANOVA was based on the model, Y_ijk_ = μ + α_i_ + β_j_ + (α*β)_ij_ + γ_k(j)_ + ε_ijk,_ where μ is the mean, α_i_ is the effect of genotype i, β_j_ is the effect of year j, (α*β)_ij_ is the interaction effect between year and genotype, γ_k(j)_ is the random effect of blocks nested within years, and ε_*ijk*_ is the residual effect. Year and genotype were used as fixed effects. The correlation between rAUDPC and mean maturity was done in R statistical software by the Spearman method (R Core Team, 2020). The broad-sense heritability of early blight resistance was estimated as H^2^ = σ^2^_g_/(σ^2^_g_ + σ^2^_yg_/y + σ^2^_e_/ry), where σ^2^_g_ = genotype variance, σ^2^_yg_ = year × genotype variance, σ^2^_e_ = error variance, r = number of replications, and y = number of years. Broad sense heritability (H^2^) and its 95% confidence interval were calculated by the SOMMER R package with Linear Mixed Model (Covarrubias-Pazaran, 2016). Maturity-corrected rAUDPC (MCR) values were calculated from the linear regression of average maturity on average rAUDPC by year with the regression procedure with R function lm() based on the general linear model, Y = βX + β_0_, where Y is the matrix of rAUDPC, X is the matrix of maturity, β is the regression coefficient and β_0_ a matrix containing errors. To distinguish genetic resistance or susceptibility from the effect of maturity, studentized residuals were examined. To keep an experiment-wise error rate of alpha = 0.05, we computed the error rate for individual progeny as: 0.05 = 1−(1−alpha_ind_)^n,^ where alpha_ind_ is the error rate for an individual progeny and n is the number of progeny. Solving for *n* = 233 (the number of progeny with full set of data), the error rate for an individual progeny was calculated as 0.0002. The *t*-value corresponding to an alpha_ind_ of 0.0002 with *n* = 233 was 3.59 (Pardoe, 2012). If the studentized residual was greater than 3.59 or less than −3.59, it indicated that the predicted value was significantly different from the observed value. If the predicted value of rAUDPC was significantly less than the observed value, the genotype was significantly more resistant to early blight than expected based on its maturity. If the predicted value of rAUDPC was significantly higher than the observed value, the genotype was significantly more susceptible to early blight than expected based on its maturity.

### Linkage Map Construction

The linkage map was constructed by “MAPpoly” R package (v. 0.2.1) following the software manual (Mollinari and Garcia, 2019). Any SNP marker with more than 20% missing data was filtered out. Any individual with more than 10% missing data was filtered out. After filtering the missing data, 233 genotypes were used for map construction, data analysis and QTL mapping. Non-redundant markers following the expected Mendelian segregation pattern at a 5% confidence level from a chi-square test were used for two-point analysis. Marker orders were estimated by both genome position and MDS (multidimensional scaling) algorithm. Then, the two maps were compared. Genome order instead of MDS was used for final map construction. The conditional probability (error = 0.05) of all possible 36 genotypes along the 12 linkage groups for all individuals in the full-sib population was estimated by the function calc_genoprob_error for QTL mapping in the next step.

### Quantitative Trait Loci Mapping

Quantitative trait loci mapping was done with the R package “qtlpoly”(v.0.2.3) with random-effect model (Pereira et al., 2020). Briefly, conditional probabilities of the linkage map from MAPpoly and phenotypes were used for QTL detection. The function “remim()” was used to search QTLs with forward stringent significance at 0.0112 level and backward elimination stringent significance at 0.0028 level. The genome-wide significance levels were estimated by the score-based resampling method, every position in the map was repeated 1000 times. Then REML (residual maximum likelihood) was used to estimate the heritability of the found QTLs (Qu et al., 2013). In this experiment, the effects of dominance and epistatic were neglected because currently there is no statistical model and/or software available for this analysis in autopolyploid plants.

## Results

### Phenotypic Variation of Parents and Full-sib Family

**FIGURE 1:**
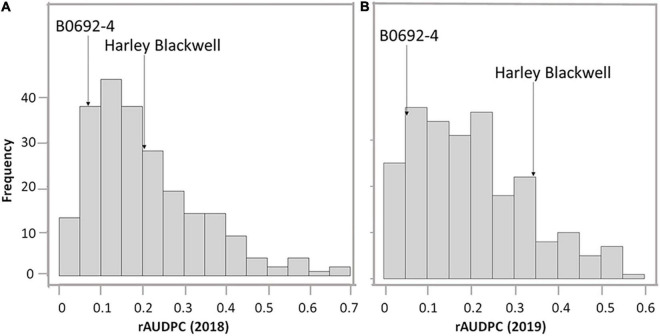
The frequency distribution of relative area under the disease progress curve (rAUDPC) of early blight among the full-sib population in 2018 **(A)** and 2019 **(B)**.

The phenotypic differences between parents as well as the full-sib family were summarized in Table 1. Harley Blackwell showed more early blight susceptibility than B0692-4 in both years by having higher rAUDPC values (Table 1). The differences between the two parents were significant at the 0.05 probability level in both years. Harley Blackwell showed earlier maturity than B0692-4 with a lower maturity value (Table 1) and the difference between the parents was significant at the 0.01 probability level. Transgressive segregation among F1 full-sib family was observed (Figures 1A,B). In 2018, the rAUDPC ranged from 0.016 to 0.679; in 2019, the rAUDPC ranged from 0.017 to 0.554. A continuous distribution of rAUDPC was observed each year. ANOVA of rAUDPC data collected in 2018 and 2019 indicated that there was a significant difference among individual genotypes in the F1 full-sib family (Table 2). When the rAUDPC data of the F1 family were compared between 2 years, there was no significant difference between the 2 years (Table 2). The rAUDPC between 2 years was moderately correlated (*r* = 0.5063, Supplementary Figure 1). This suggested that the levels of susceptibility or resistance of F1 genotypes to early blight were consistent in both years in general although the interaction between genotype and environment was significant at the 0.01 probability level (Table 2).

**TABLE 1:** The measurements of the traits in the full-sib family and the parents in 2018 and 2019.

Trait	Harley Blackwell	B0692-4	Mean of full-sib family	Range of full-sib family
rAUDPC	0.2011*| | ^b^0.3459*	0.0785*| | 0.0527*	0.2076| | 0.2029	0.0164–0.6788|| 0.0170–0.5539
Maturity^a^	2.3**	5.5**	3.1	1–5.5

*^a^Data collected from 2018 and 2020 in Maine, the values are the mean of 2 years data.*

*^b^The number of the left side of “||” is the data of 2018; the number of the right side of “||” is the data of 2019.*

*Indicated significant difference between parents at p = 0.05 level by t-test in2018 and 2019, respectively.

**Indicated significant difference between Harley Blackwell and B0692-4 at p = 0.01 level by t-test.

**TABLE 2:** ANOVA of the relative area disease progress curve (rAUDPC) among the full-sib population.

Trait	Variation	DF	Mean square	*F* Value	Pr(>F)	H^2a^
rAUDPC	Year	1	0.0107	0.08	0.8476	0.732 ± 0.035
	Block within year	2	0.1342	12.66	<0.0001	
	Genotype	234	0.0680	6.4	<0.0001	
	Year:Genotype	229	0.0197	1.85	<0.0001	
	Error	460	0.0103			

*^a^H^2^ indicates broad sense heritability.*

### Correlations Between Traits

**FIGURE 2:**
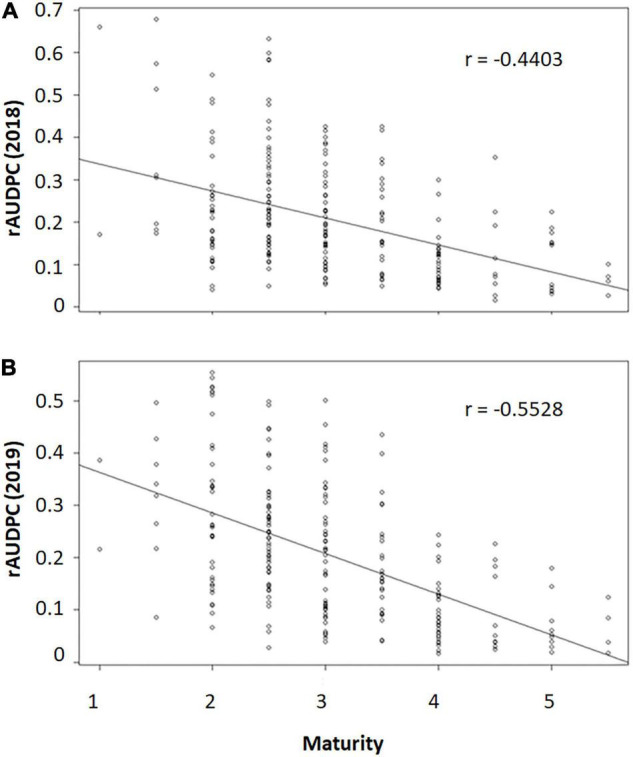
The scatter plots of maturity and relative area under the disease progress curve (rAUDPC) of early blight in 2018 **(A)** and 2019 **(B)**. *r* value indicating the spearman’s rank correlation coefficient. 1 = very early maturity; 2 = early maturity; 3 = medium early maturity; 4 = medium late maturity; 5 = late maturity; and 6 = very late maturity.

The rAUDPC in both years were moderately negatively correlated with maturity (r_2018_ = −0.4403, r_2019_ = −0.5528, Figures 2A,B). This suggested that genotypes with late maturity tended to be more resistant to early blight than genotypes with early maturity. To further dissect the genetic components independent of maturity, studentized residuals were used to find out the outliers based on the prediction of maturity. The results indicated that there were no offspring significantly more susceptible or resistant than expected based on maturity in both 2018 and 2019 (Supplementary Tables 1, 2).

### Broad Sense Heritability of Early Blight Resistance

As an important potato breeding parameter, broad sense heritability of rAUDPC was estimated as 0.73 with a 95% confidence interval from 0.66–0.80 (Table 2). However, there was a significant interaction between genotype and environment (Table 2). It accounted for 17.6% variation in the total phenotypic variation.

### The Linkage Map Construction

About 20k SNP markers were used to genotype 241 full-sib progenies and two parents. With eliminating the redundant markers and filtering F1 plants with more than 10% missing data, the linkage map was based on 9124 SNP markers and 233 full-sib F1 plants (Table 3 and Supplementary Table 3). The map covered the length of 1469.34 cM with an average of 0.16 cM intervals between two adjacent markers. The max gaps on each chromosome are between 2.31 and 8.01 cM.

**TABLE 3:** Summary of the linkage map from a cross between Harley Blackwell and B0692-4.

Chr	Map size (cM)	Markers/cM	Simplex	Double-simplex	Multiplex^a^	Total	Max gap
1	173.61	6.86	310	201	680	1191	8.01
2	170.49	5.06	254	124	484	862	4.10
3	136.57	5.97	199	116	500	815	5.98
4	123.10	6.43	269	133	388	790	3.49
5	111.69	7.66	348	87	421	856	2.82
6	139.77	4.91	160	117	409	686	4.94
7	109.31	6.72	279	80	376	735	4.94
8	85.88	7.13	146	87	379	612	4.04
9	98.15	6.74	224	129	309	662	2.31
10	123.15	4.85	167	92	338	597	5.39
11	109.35	6.63	194	136	395	725	2.33
12	88.27	6.72	123	97	373	593	2.37
Total	1469.34	6.24	2673	1399	5054	9126	5.39

*^a^Multi-dose.*

### Quantitative Trait Loci for Early Blight Resistance

**FIGURE 3:**
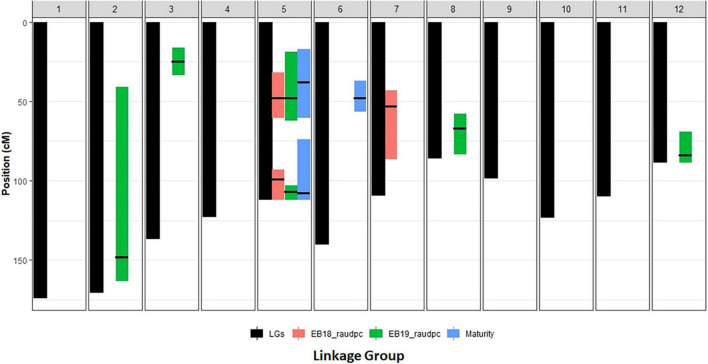
The presentation of QTLs positions of early blight resistance and maturity on linkage map from the cross between Harley Blackwell and B0692-4.

Quantitative trait locus detected for early blight resistance were listed in Table 4, Figure 3 and Supplementary Figures 2, 3. There were three QTLs detected in 2018. Two QTLs were located on chromosome 5 and another one was located on chromosome 7. There were six QTLs detected in 2019. The two major QTLs were detected on chromosome 5, and others were detected on chromosomes 2, 3, 8, and 12, respectively. In 2018, two QTLs on chromosome 5 explained 32% of the phenotypic variation, and one minor QTL on chromosome 7 explained 8.5% of the phenotypic variation. In 2019, two QTLs on chromosome 5 explained 45% of the phenotypic variation, while the other four minor QTLs totally explained 25% of the phenotypic variation.

**TABLE 4:** QTLs for early blight foliar resistance and foliar maturity from a full-sib population derived from Harley Blackwell and B0692-4.

Trait	Chr	Position (cM)	Marker	h^2a^	QTL code
Maturity	5	38	PotVar0079803	0.3962	MT-5.1
	5	108	PotVar0034819	0.1204	MT-5.2
	6	48	solcap_snp_c2_31648	0.0711	MT-6
Sum				0.5878	
EB18_rAUDPC	5	48	solcap_snp_c2_38140	0.1966	EB-5.1
	5	99	PotVar0123189	0.1274	EB-5.2
	7	53	solcap_snp_c2_25212	0.0854	EB-7
Sum				0.4094	
EB19_rAUDPC	2	148	solcap_snp_c1_12771	0.0712	EB-2
	3	25	solcap_snp_c2_41061	0.0575	EB-3
	5	49	solcap_snp_c2_45539	0.2698	EB-5.1
	5	107	PotVar0034819	0.1800	EB-5.2
	8	67	PotVar0081130	0.0533	EB-8
	12	84	solcap_snp_c2_6466	0.0658	EB-12
Sum				0.6977	
MCR18^b^	8	2	solcap_snp_c2_6466	0.1393	MCR-8
MCR19	5	25	PotVar0025046	0.1533	MCR-5

*^a^Heritability of each QTL locus.*

*^b^Maturity corrected resistance.*

### Quantitative Trait Loci for Maturity

Quantitative trait locus for maturity were detected with data collection from 2 years. The results were listed in Table 4, Figure 3, and Supplementary Figure 4. One major QTL was detected on the short arm of chromosome 5. Two minor QTLs were detected on the long arm of chromosome 5 and 6, respectively. The major QTL on chromosome 5 explained 40% of the phenotypic variation. The two minor QTLs together explained about 19% of the phenotypic variation.

### Quantitative Trait Loci for Maturity Corrected Resistance

Quantitative trait locus for MCR were listed in Table 4 and Supplementary Figures 5, 6. One QTL was detected on chromosome 8 in 2018. One QTL was detected on chromosome 5 in 2019. The MCR QTL detected in 2018 explained 13.9% of the phenotypic variation. The MCR QTL detected in 2019 explained 15.3% of the phenotypic variation.

### Comparison Between Locations of Quantitative Trait Locus for rAUDPC and Maturity

Two QTLs for maturity were detected on the short arm of chromosome 5 at 38 cM and the long arm at 108 cM, respectively. In 2018, two QTLs for early blight resistance were detected on chromosome 5 at similar locations to maturity QTLs (48, 99 cM). In 2019, two major QTLs for early blight resistance were detected at similar locations to maturity QTLs on chromosome 5 at 49 and 107 cM. It suggested that these two QTLs for maturity and two QTLs for early blight resistance might be closely linked. Other QTLs showed no overlap between early blight resistance and maturity. One QTL for MCR on chromosome 5, shared the overlapped position with the resistance and maturity QTLs on the short arm of chromosome 5. The position of this QTL was shifted to the position at 25 cM. Another QTL for MCR detected in 2019 on chromosome 8 was a new QTL. It did not overlap with other QTLs.

## Discussion

In this study, a full-sib family population with 241 F1 progeny derived from a cross between a durable early blight resistant clone, B0692-4, and a susceptible cultivar, Harley Blackwell, was used for early blight disease resistant genetic dissection. B0692-4 shows durable early blight resistance in recent years (data not shown). B0692-4 was used as the paternal parent of the cross and the resistance from the cytoplasm cannot be dissected in this study. High density SNP array used in this study can provide better coverage of the whole genome and smaller gaps between two adjacent makers. It will also provide plentiful makers in the QTL fine mapping stage. The total length of the genetic linkage map in this study is longer than in previous studies (Zhang, 2005; Odilbekov et al., 2020). This difference might come from two aspects. The main one is that our SNP markers were across the whole potato genome, providing more coverage than the traditional DNA markers or old SNP marker sets. Another one is that SNP array data could carry some errors and it can make the linkage map longer than its natural length. In this population, we found a continuous distribution of rAUDPC and transgressive segregation for early blight resistance among the F1 family. We elucidated the genetic nature of early blight resistance in this population and provide insight for early blight resistant breeding in the future.

### Heritability of Early Blight Resistance in the Population

Resistance to early blight is a complex quantitative genetic trait since it is controlled by multiple genes (Zhang, 2005; Odilbekov et al., 2020). In our study, we did not observe any vertical resistance in the full-sib population. This finding is in agreement with previous studies in both tetraploids and diploids or wild relatives (Herriott et al., 1986; Boiteux et al., 1995; Christ and Haynes, 2001; Duarte et al., 2014; Haynes and Qu, 2016; Abuley et al., 2018; Xue et al., 2019). The phenomenon of transgressive segregation in both directions (resistance and susceptibility) was found in our population. In our QTL mapping result, a few alleles from the susceptible parent, Harley Blackwell, contributed to the QTL associated with resistance. This indicates that additive genes involved in resistance from both parents were combined in the F1 population. This phenomenon was also found in another population (Odilbekov et al., 2020). All these findings suggest that multiple QTLs contribute to early blight resistance in our population.

Broad sense heritability is a useful tool to assess the potential heritability of quantitative traits. In this study, broad sense heritability was estimated as 0.73 with a 95% confidence interval of 0.66–0.80. In a recent similar study, Odilbekov et al. (2020) estimated broad sense heritability as 0.48 in a full-sib tetraploid population with 80 F1 progeny. Zhang (2005) estimated broad sense heritability as 0.77 with a 95% confidence interval of 0.71 to 0.82 in a 219 full-sib diploid family. In our previous study, broad sense heritability was estimated higher as 0.89 with a 95% confidence interval of 0.86 to 0.92 in 217 potato cultivars (Xue et al., 2019). In this study, the interaction between genotype and environment is much higher than in our previous study. It accounted for 17.6% of the total variation in this study while it only accounted for 7.8% of the total variation in the potato cultivar population. Overall, early blight resistance is heritable although there are variations in the heritability among different germplasm and there were significant interactions between genotype and environment.

In this study, we also estimated the heritability of each QTL locus. In 2018, the total detectable heritability of QTLs was 0.4094; in 2019, the total detectable heritability of QTLs was 0.6977. This result is similar to the previous study with the family-based narrow sense heritability from 0.64 to 0.78 in diploid potatoes (Ortiz et al., 1993). According to these results, more than 40% of early blight resistance is additive genetic variance, thus, marker-assisted selection could be useful for early blight resistance potato breeding.

### Genetic Basis of Early Blight Resistance Varies in Different Populations

The genetic basis of early blight resistance is poorly understood. Zhang (2005) studied early blight resistance in a diploid hybrid family of 219 genotypes derived from *S. phureja* × *S. stenotomum*. Five QTLs for early blight resistance were detected on chromosomes 4, 5, 9, 11, and 12, respectively. Together these QTLs accounted for 62.2% of the total phenotypic variation. Two QTLs on chromosome 4 and chromosome 5, respectively, were mapped to the same positions as QTLs for maturity. These two QTLs were considered as maturity-related early blight resistance, which explained 29.2% of the total phenotypic variation. QTLs for early blight resistance unrelated to maturity (“true” resistance) accounted for 33% of the total phenotypic variation for early blight resistance. QTL on chromosome 9 was a major QTL that explained 18.3% of the early blight resistance variation in this population. Recently, QTLs for early blight foliar resistance were detected on chromosomes 1, 5, 6, 7, 10, 11, and 12 in a tetraploid potato population (Odilbekov et al., 2020). The QTLs on chromosomes 5 and 11 for foliage resistance to early blight were independent of foliar maturity. The QTL on chromosome 11 accounted for more than 50% of the phenotypic variation.

In our study, three QTLs for early blight resistance were detected in 2018. The two QTLs on chromosome 5 explained 19.7 and 12.7% of the phenotypic variation, respectively. The QTL on the short arm of chromosome 5 showed a similar position to the QTL found in previous research (Zhang, 2005; Odilbekov et al., 2020). However, the QTLs on the long arm of chromosome 5 and on chromosome 7 were not detected in previous research. In 2019, except for QTLs on chromosome 5, four other minor QTLs were detected, but the QTL on chromosome 7 was not detected. These four minor QTLs found on different chromosomes do not share similar positions with QTLs for maturity.

Besides maturity-related resistant QTLs being found in common between our study and other studies (Zhang, 2005; Odilbekov et al., 2020), there is no major “true” resistant QTL (not related to maturity) found in both years in our population. While in the other two studies, major QTLs unrelated to maturity were detected on chromosome 9 and chromosome 11, respectively (Zhang, 2005; Odilbekov et al., 2020). In our population, these six minor QTLs unrelated with maturity together explained 34% of the phenotypic variation in 2019. There was only one “true” resistant QTL detected on chromosome 7 in 2018 which explained 8.5% of the phenotypic variation. These differences between our study and other studies might come from populations derived from different parents. Also, the linkage maps were incomplete in the other two studies, and there were only 80 F1 plants in the research from Odilbekov et al. (2020), potentially increasing the chance of finding ghost QTLs and reducing the chance of finding true QTLs.

Although the analysis of the residuals found that the early blight resistance value of all of the genotypes was as expected based on their maturity, we still found minor maturity unrelated QTLs detected in both years, and one MCR QTL in 2019. This difference could come from different statistical methods and/or the choice of levels of stringent significance. Interestingly, Odilbekov et al. (2020) showed the result of high correlation (*r* = −0.85) between relative area under the defoliation curve and rAUDPC, but they still detected a major QTL for early blight resistance unrelated with foliage maturity.

In our result, we detected three maturity QTLs. The major QTL on the short arm of chromosome 5 explained 39.6% of the total variation. This major QTL could be the *StCDF1* gene which was located at a similar position contributing to potato maturity (Kloosterman et al., 2013). The QTL on the long arm of chromosome 5 and the QTL on chromosome 6 were not reported in previous research. These two minor QTLs explained 12.0 and 7.1% of the total variation, respectively. The QTL of chromosome 6 is not related to early blight resistance.

### Improvement of Early Blight Resistance in Potato Breeding Program

Our results may have potential implications in potato breeding programs. SNPs linked with QTLs can be used for selection for early blight resistance. In our mapping results, all QTLs unrelated to maturity were minor QTL; the heritability (h^2^) of these QTLs ranged from 5.3 to 8.5%. Phenotypic selection would be inefficient to incorporate the genetic resistance from minor QTLs. Developing DNA markers could speed up the process of breeding early blight resistant potatoes. However, manipulation of multiple QTLs can be a challenge (Ribaut and Hoisington, 1998). For major QTLs, marker-assisted selection would be a good choice for plant breeding. However, for minor QTLs, genome selection would be a better choice to increase the genetic gain of complex traits. In a previous early blight resistant potato breeding program, Christ and Haynes (2001) found that early blight resistance was a highly heritable trait. However, after one cycle of maternal half-sib recurrent selection for early blight resistance based on phenotypic selection, they ended up with a population that was later maturing and more susceptible to early blight than the starting population (Cruz et al., 2009).

On the other hand, lateness is not a desirable trait in potatoes. In previous studies, most resistant cultivars or breeding clones were late or medium-late in maturity (Boiteux et al., 1995; Xue et al., 2019). Jansky and Rouse (2003) successfully found two early blight resistant clones without late maturity in their 32 interspecific *Solanum* hybrid clones. It is therefore worthwhile to mine new germplasm resistant to early blight from wild *Solanum* germplasm. With the markers linked with early blight resistant QTLs mapped in the present study, it would help to identify new alleles which could contribute more resistance than that found in our population. However, the interaction between genotype and environment should be considered carefully.

## Conclusion

In this study, we identified three early blight resistant QTLs in 2018 and six early blight resistant QTLs in 2019. The QTL on chromosome 7 identified in 2018 and four minor QTLs identified in 2019 were unrelated to maturity. The results indicated that the minor QTLs unrelated with late maturity together can contribute a similar resistance level as that from QTLs related to late maturity. The QTLs identified in this study can be used in a breeding program or as the starting point for gene isolation.

## Data Availability Statement

The original contributions presented in the study are included in the article/Supplementary Material, further inquiries can be directed to the corresponding author.

## Author Contributions

WX, KH, and XQ designed the study. KH generated the full-sib population and collected maturity data. WX and XQ collected the early blight resistant phenotypic data. WX performed data analysis and wrote the manuscript. KH, CC, and XQ revised the manuscript. All authors read and approved the final manuscript.

## Conflict of Interest

The authors declare that the research was conducted in the absence of any commercial or financial relationships that could be construed as a potential conflict of interest.

## Publisher’s Note

All claims expressed in this article are solely those of the authors and do not necessarily represent those of their affiliated organizations, or those of the publisher, the editors and the reviewers. Any product that may be evaluated in this article, or claim that may be made by its manufacturer, is not guaranteed or endorsed by the publisher.
