# Supplementary figures and images for: Genetic Dissection of Early Blight Resistance in Tetraploid Potato
Source: Front Plant Sci. 2022 Mar 25;13:851538. doi: 10.3389/fpls.2022.851538 (PMC8990756; doi:10.3389/fpls.2022.851538)

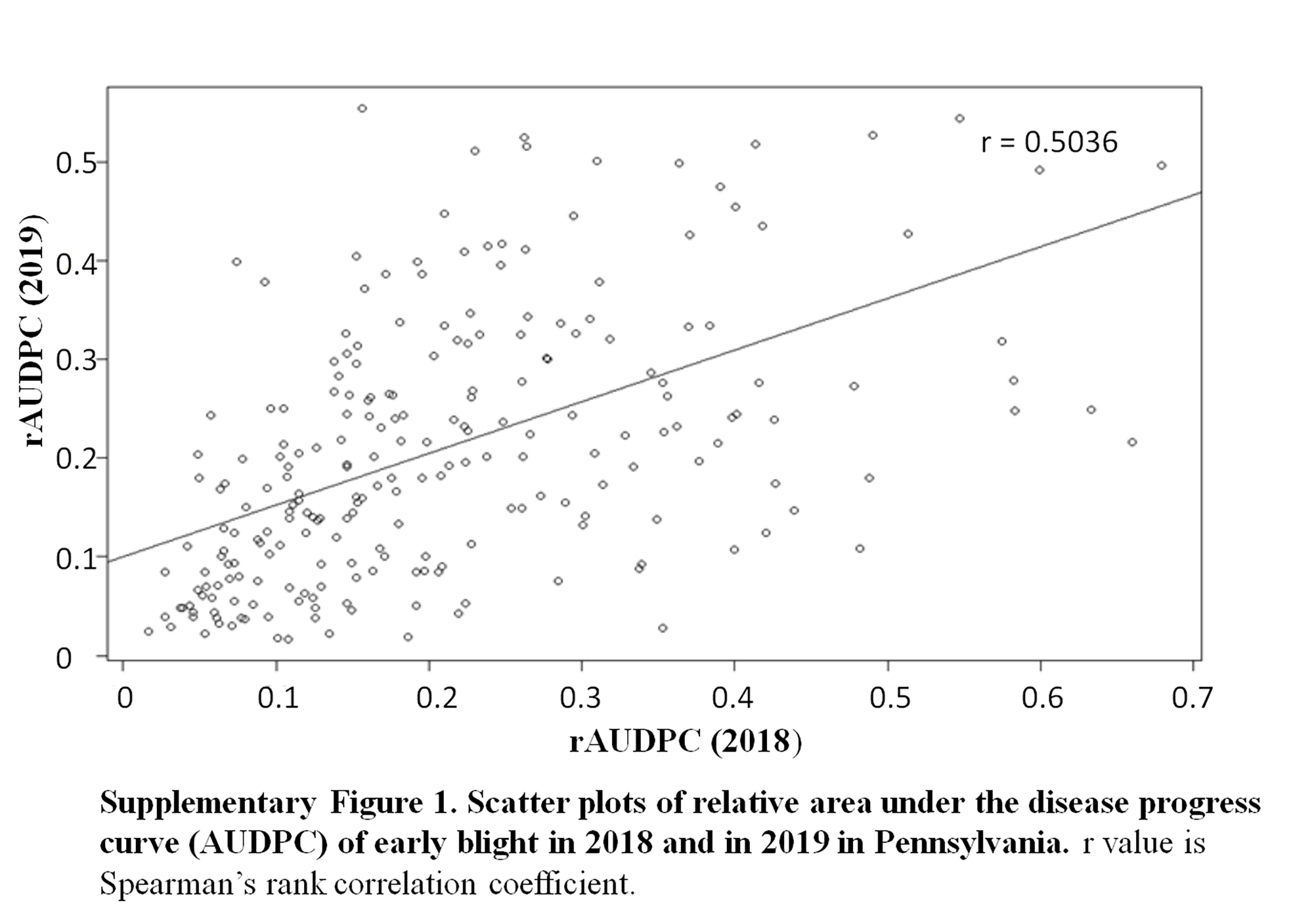

Supplement: Supplementary file 1 [file Image_1.JPEG]

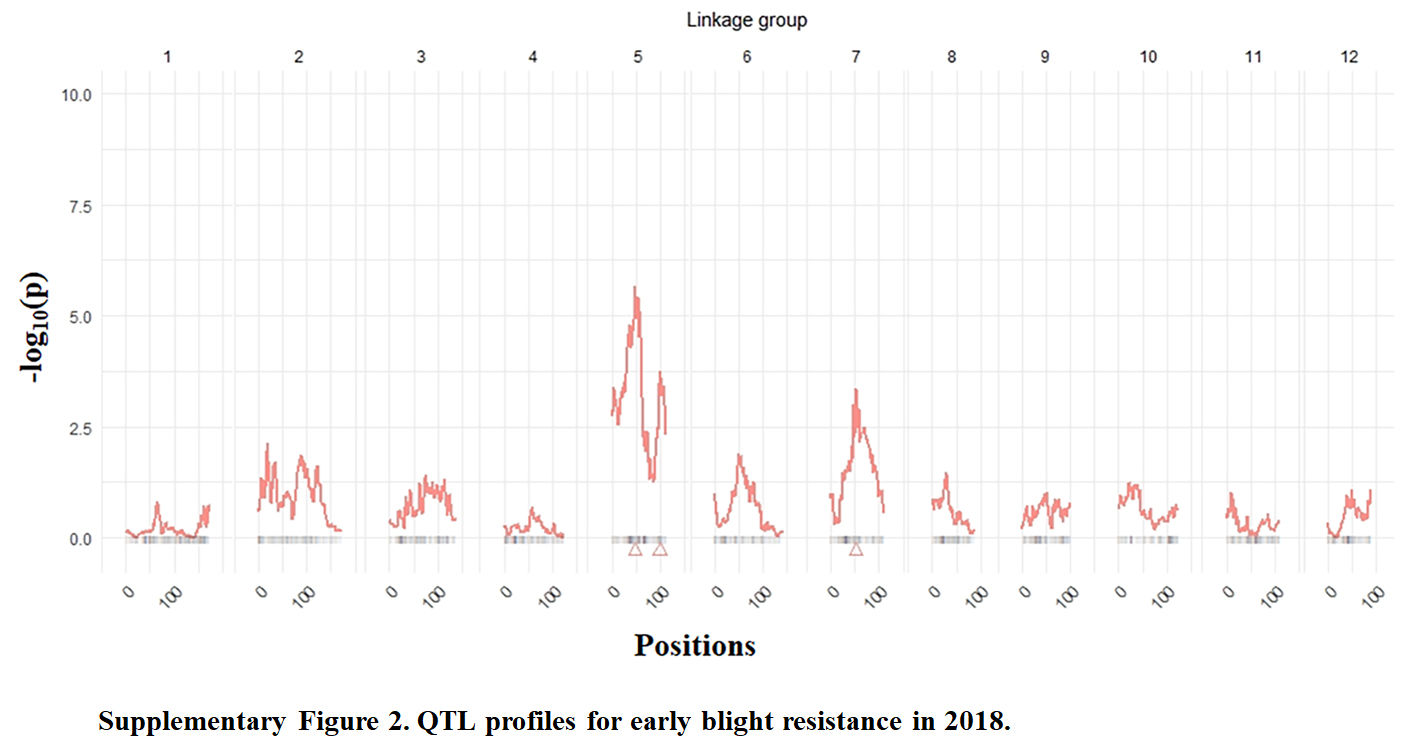

Supplement: Supplementary file 2 [file Image_2.JPEG]

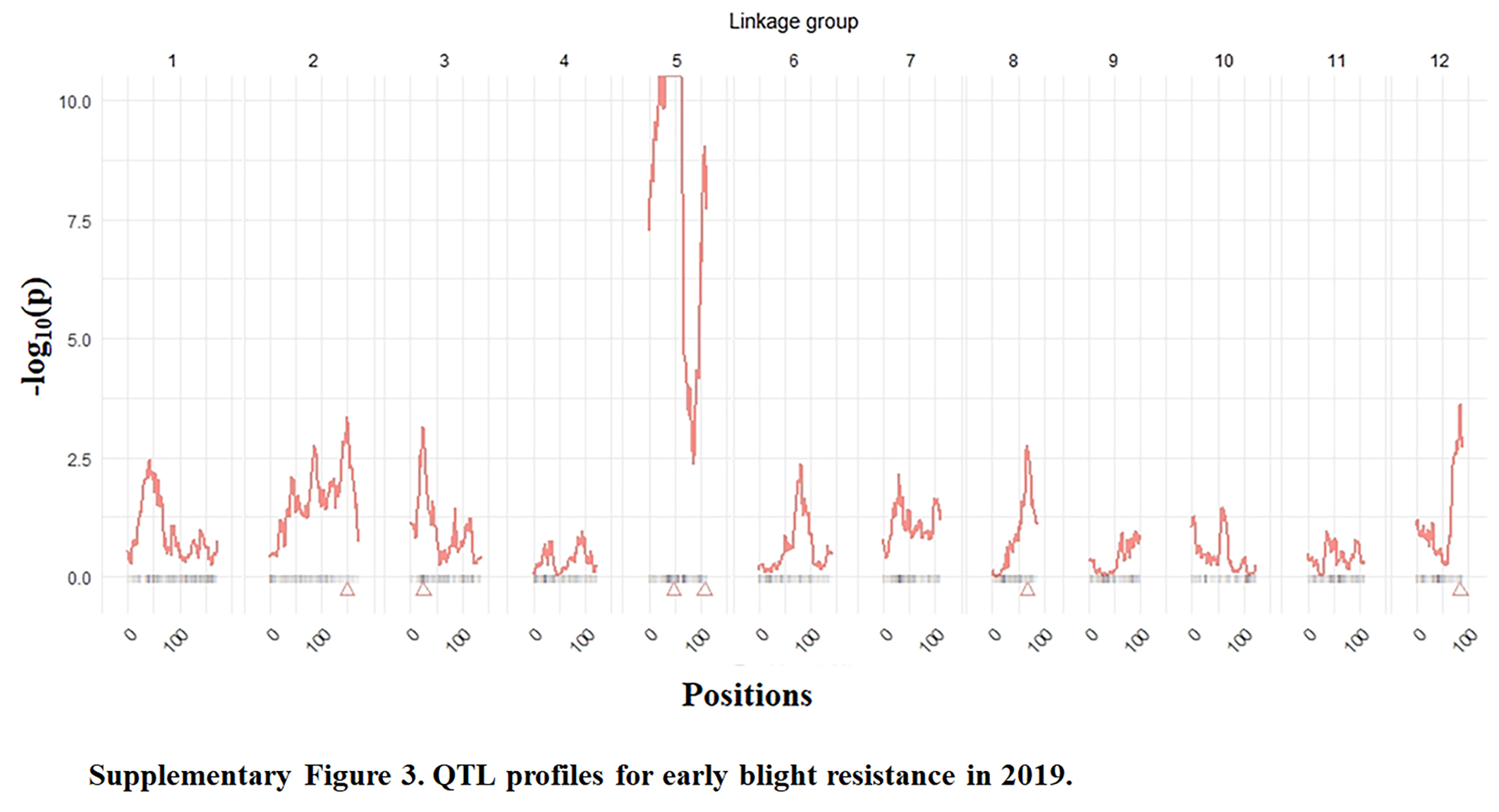

Supplement: Supplementary file 3 [file Image_3.JPEG]

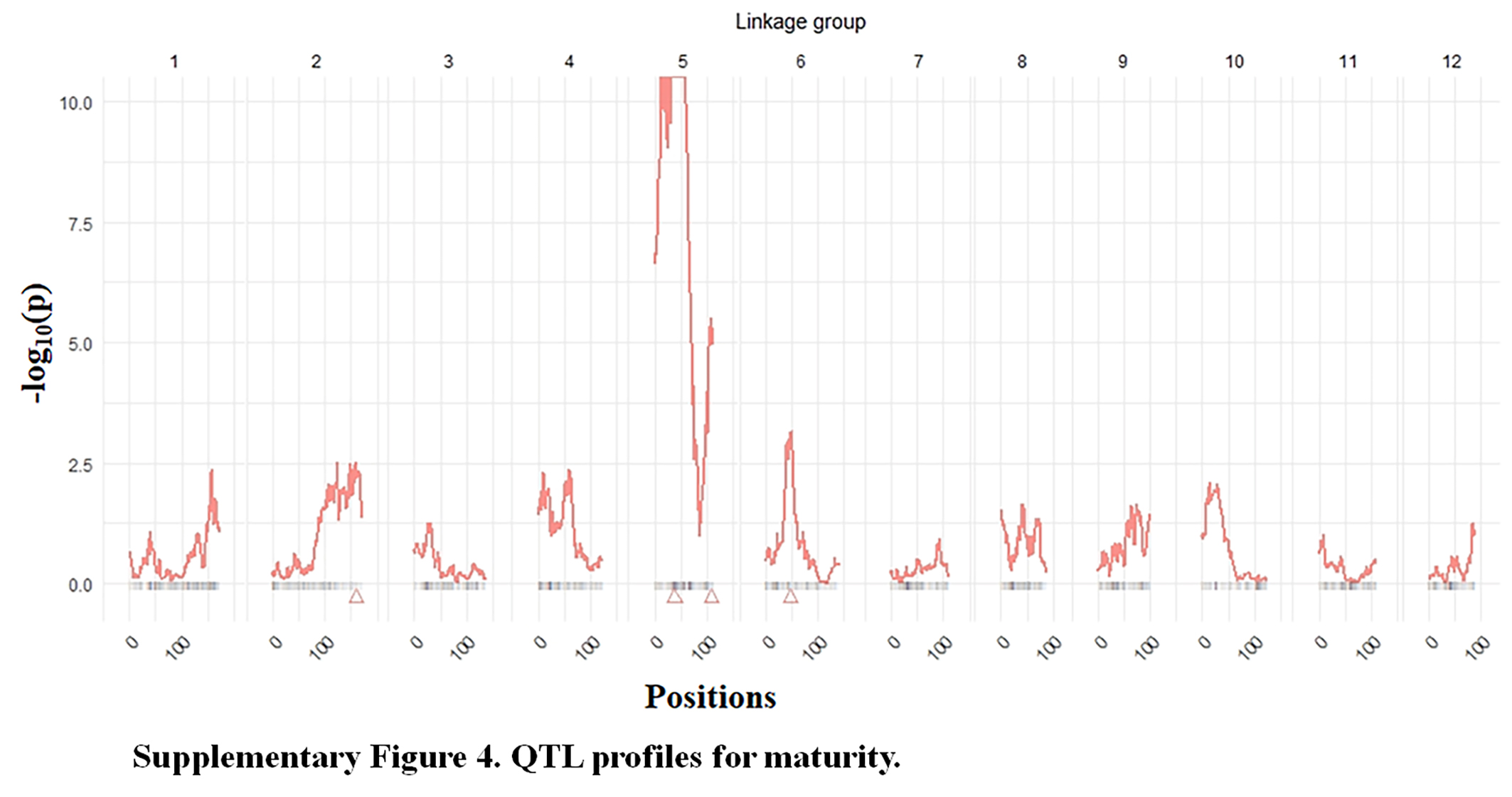

Supplement: Supplementary file 4 [file Image_4.JPEG]

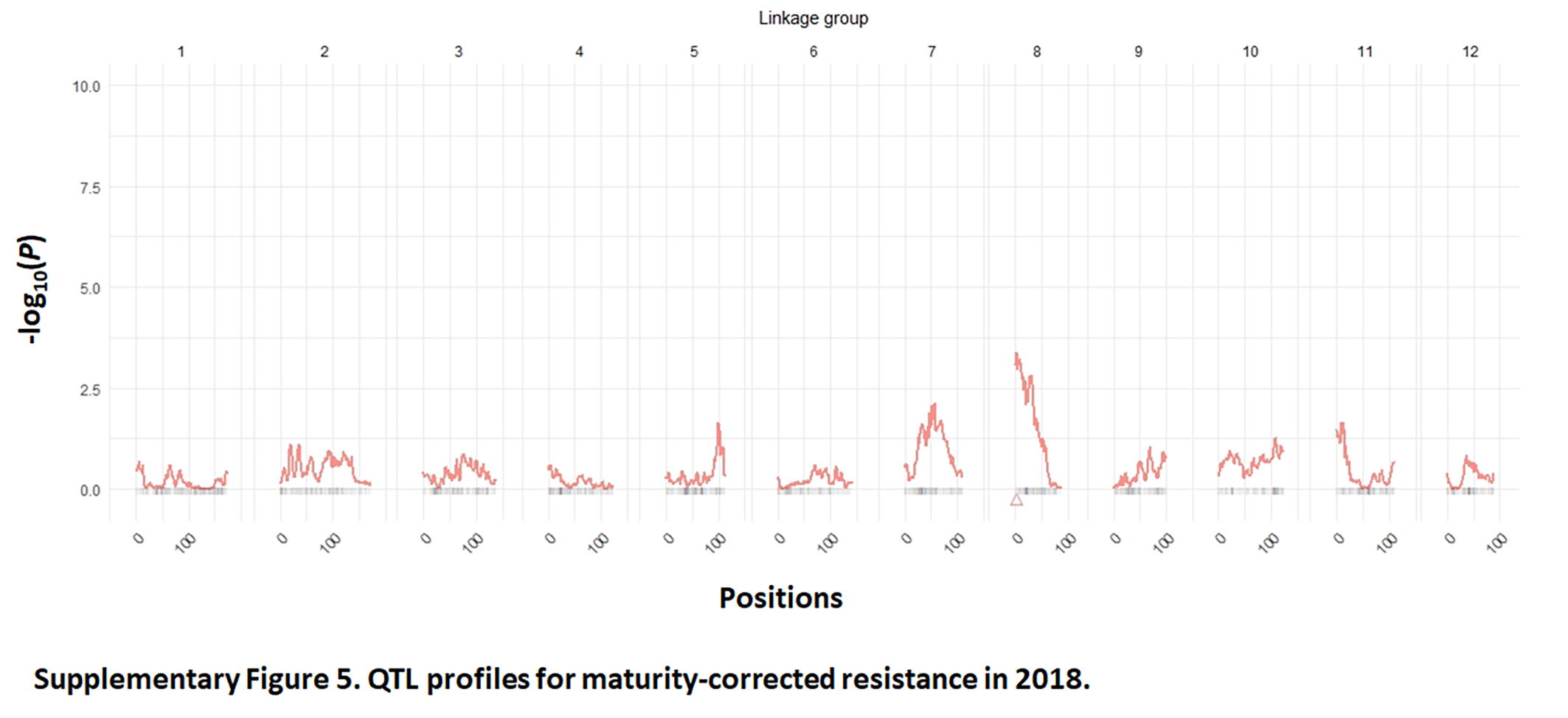

Supplement: Supplementary file 5 [file Image_5.JPEG]

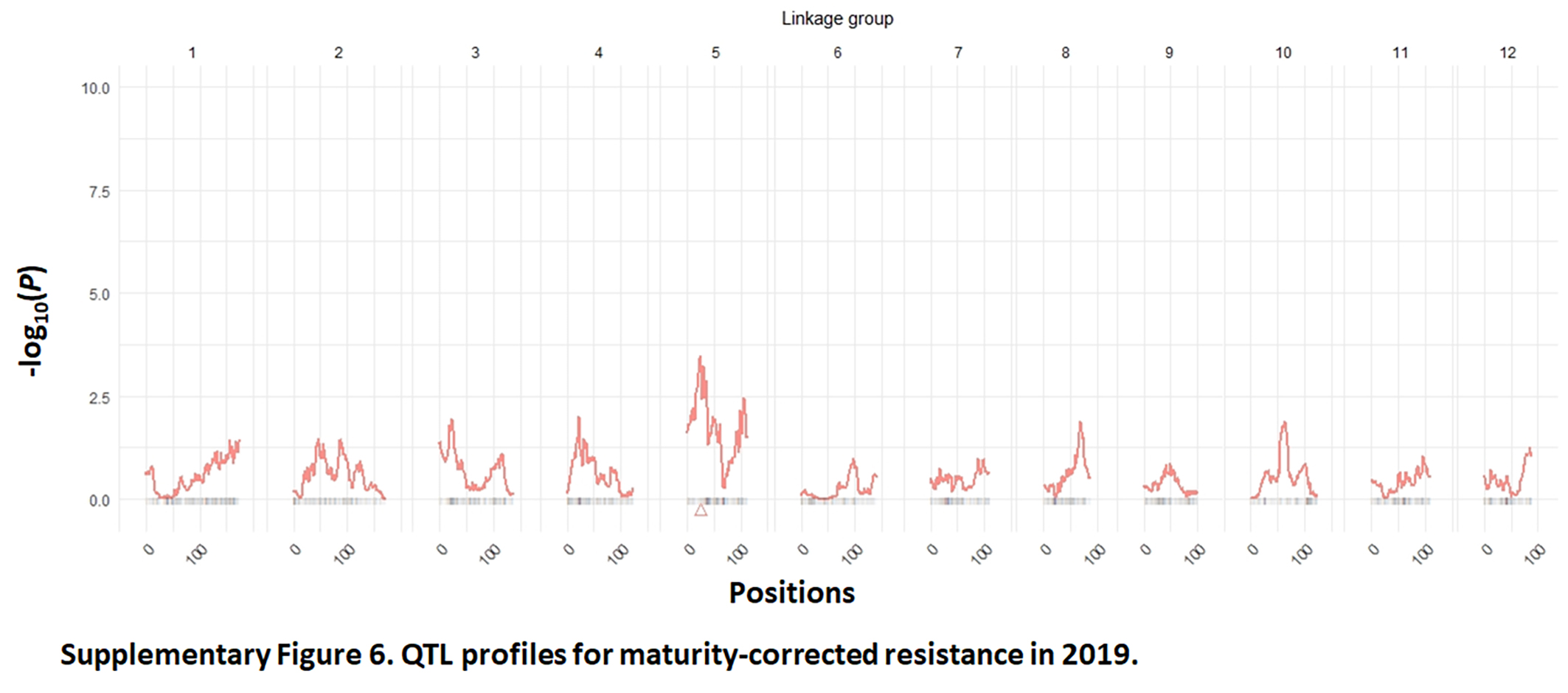

Supplement: Supplementary file 6 [file Image_6.JPEG]
